# Antibacterial Treatment of Selected High-Touch Objects and Surfaces within Provision of Nursing Care in Terms of Prevention of Healthcare-Associated Infections

**DOI:** 10.3390/healthcare9060675

**Published:** 2021-06-04

**Authors:** Martin Krause, František Dolák

**Affiliations:** 1 Institute of Nursing, Midwifery and Emergency Care, Faculty of Health and Social Sciences, University of South Bohemia, 37001 České Budějovice, Czech Republic; fdolak@zsf.jcu.cz; 2 Institute of Nursing and Emergency Care, Faculty of Health Studies, Technical University of Liberec, 46117 Liberec, Czech Republic

**Keywords:** antimicrobial nanolayer, bacterial contamination, prevention, healthcare-associated infections, nursing, high-touch objects and surfaces

## Abstract

Prevention of healthcare-associated infections is an important part of providing nursing care. High-touch objects and surfaces that can be contaminated with various bacteria are matters of concern. The possibility of reducing contamination is the use of antibacterial and hydrophobic nanolayers. The aim of this study was to determine, by means of an experimental method, the microbial efficacy of applied antibacterial and hydrophobic nanolayers on high-touch objects and surfaces used in nursing practice in a regional hospital in the Czech Republic. The results show that the antibacterial efficacy of the applied nanolayer was not demonstrated. Furthermore, the results show that selected objects and surfaces can always be contaminated by bacterial agents in about 1/3 of cases. It is mainly contamination with nonpathogenic bacteria; however, the presence of pathogenic bacteria, such as *Staphylococcus aureus*, has also been detected. The results of this study pinpoint the importance of following the basic rules for the use of decontaminated objects and surfaces used to provide healthcare.

## 1. Introduction

Healthcare-associated infections are still a current problem and, according to the Organization for Economic Co-operation and Development, they are among the most common adverse events in provision of healthcare services [1,2]. Healthcare-associated infections significantly affect patient mortality and morbidity, including increased financial costs of healthcare [3]. According to the European Parliament, an average of 1 in 20 patients acquire healthcare-associated infection every day in the European Union, i.e., 4.1 million patients and 37,000 patients die from healthcare-associated infections. At the same time, it is estimated that 20–30% of infections could be prevented [4]. In many cases, healthcare-associated infections can be effectively prevented using evidence that can reduce the incidence of these infections. One of the basic components of prevention is the observance of basic hygienic-epidemiological principles, including the decontamination of objects and surfaces intended for re-use [5].

Provision of healthcare is closely linked to the use of a variety of objects and surfaces, such as medical equipment and medical devices, including tools, instruments, gadgets and other objects [6]. Objects and surfaces of the hospital environment can be a potential reservoir for the transmission of bacteria and other microorganisms immediately after contaminated hands [7]. Transmission through contaminated hands or contact with contaminated surfaces may contribute up to 20–40% to the development of healthcare-associated infections [8,9,10]. According to available research, surfaces are a frequent source of transmission of pathogens associated with healthcare through direct contact with the patient or the environment, or indirect contact through contaminated hands of healthcare professionals, including nurses. Noncritical objects and surfaces are based on the Spaulding classification, which categorizes reusable medical devices for decontamination [11,12]. The matter of concern is mainly noncritical objects and surfaces, which are associated with frequent hand contact [13,14]. In terms of contact frequency, objects and surfaces can be divided into high-touch (e.g., stethoscopes, telephones, medical records) and low-touch or minimal-touch (e.g., ceilings, mirrors, walls) [6,8,12,15,16]. Another important aspect of the transmission of agents is the ability of microorganisms to persist on dry and inanimate surfaces, where the main principle for reducing cross-transmission is the observance and implementation of regular decontamination of objects and surfaces [12,14,17]. High-touch objects and surfaces, which are used to provide healthcare services and specific nursing interventions, require increased attention since they are directly involved in the microorganism transmission chain. For this reason, they require cleaning and subsequent disinfection [18,19]. The Spaulding classification can be used to select the method of decontamination of individual objects and surfaces intended for repeated use [6,20]. In practice, the decontamination of high-touch objects and surfaces might be underestimated, as healthcare professionals may not be aware that these objects and surfaces could be involved in the transmission of pathogens from healthcare-associated infections [19].

Preventive measures also include finding and implementing other options, such as antimicrobial surfaces, which are used to cover or impregnate surfaces in various surfaces of healthcare provision [20]. The main goal of antimicrobial surfaces is to minimize the presence of microbial contamination or reduce the viability of organisms and reduce biofilm formation. Antimicrobial surfaces are gaining more attention for setting other infection prevention strategies [21,22]. The need for antimicrobial surfaces is important because bacterial contamination of the surface contributes to the transmission of healthcare-associated infections in situations where decontamination has not been performed effectively [23,24]. When antimicrobial surfaces are used on high-touch and reusable items, it has the potential to reduce the incidence of healthcare-associated infections [23]. Antimicrobial surfaces can be placed in two categories, antiadhesive surfaces (for example applying a layer of polyethylene glycol or diamond-like carbon) and antimicrobial coatings (organic antimicrobials, such as ionized silver or copper) [22]. Nanomaterials can also be used in surface treatments since there has been increasing evidence that nanomaterials offer new ways to design antimicrobial surfaces [21]. Nanoparticles of silver and other elements, which are gradually released into the environment, are important components for use in healthcare [25,26]. In particular, silver nanoparticles are one of the most important ones due to their bactericidal, fungicidal and virucidal activity [26]. At present, there are various surface treatments of objects and surfaces using a nanolayer containing various organic–inorganic particles ensuring antimicrobial efficiency. The nanolayer can be applied to objects and surfaces in various ways, e.g., by the sol-gel method [27]. Ongoing research on this surface is still important [22]. The aim of this study was to investigate the bacterial contamination of touch objects and surfaces in a nursing practice and to compare contaminations on objects covered by a nanolayer to control objects. Studies on the contaminations of objects and surfaces are important and it is necessary to develop relevant views of bacterial transmission in healthcare facilities and to highlight effective solutions to fight it.

## 2. Materials and Methods

### 2.1. Study Design

The research was carried out by the technique of experiment. Its aim was to determine the microbial effectiveness of the applied antibacterial and hydrophobic nanolayer on selected objects and surfaces in nursing practice. The research was carried out at clinical workplaces (standard surgical departments) of a regional hospital in the Czech Republic.

### 2.2. Selection of Research Samples

The research samples were selected high-touch objects and surfaces that nurses use to provide nursing care. Research samples were selected based on a previous study [28]. These were emesis basins, trays and boxes intended for storing medical supplies, including sterile material in a protective package. Two groups were chosen for the experiment: nano and control. The research samples (emesis basins, trays and boxes) were identical. The nano group included research samples to which an antibacterial and hydrophobic nanolayer was applied. The control group included research samples that were not treated with anything, i.e., without surface treatments. The research samples were selected based on the relevance and feasibility of nanomaterial application. There were six criteria for the selection of samples. Firstly, they had to be objects and surfaces that the nurses used to provide nursing care. Secondly, that objects and surfaces had to be intended for re-use. Thirdly, in order to be able to apply antibacterial and hydrophobic nanolayer. Fourthly, that objects and surfaces had not come into direct contact with the patient. Fifthly, objects and surfaces had to be high-touch. The last criterion was affordability. The control group included a total of 11 emesis basins, 12 trays and 3 boxes for medical supplies. The nano group included a total of 13 emesis basins, 12 trays and 3 boxes for medical supplies. The samples from the nano and control group were ready for use.

### 2.3. Preparation and Application of the Nanolayer

The nanolayer was applied to the entire surface of the research samples, not only to a part of it. A nanolayer (antibacterial sol), which was composed of a hybrid organic-inorganic sol based on 3-(trimethoxysilyl) propyl methacrylate with silver, copper and zinc cations, under the designation AD30, was applied to the nano research samples. The antibacterial sol corresponded to the specification according to patent CZ303861 [29]. The nanomaterial was prepared and applied by the author of the patent. Further, dilution was performed to a suitable concentration for application using isopropyl alcohol. The actual application of the antibacterial sol to the surface of plastic materials was carried out by high-pressure spraying with compressed air guns. Heat treatment was then performed to complete the polymerization of the base matrix at a temperature of 80 °C for 3 h.

### 2.4. Sampling Plan and Technique

Research samples were placed at each workplace. Each research sample was marked with an original and random code so that the result was not affected (i.e., blinding the sample origin also to the person responsible for the microbial analysis). Swabs were taken and microbiological verification of the effectiveness of the applied nanolayer was performed in the accredited microbiological laboratory of the regional type hospital every 7 days for 12 weeks. Under standard procedures and according to the conditions of laboratory practice in the Czech Republic [30,31], swabs were taken from individual research samples (ready for use for the provision of nursing care) from the area of 10 × 10 cm using a template. Sterile cotton-tipped applicator sticks moistened with saline were used for the collection. Sampling was performed using aseptic techniques. In one day, swabs were taken from all research samples. Subsequently, the sampling kit was marked and transported in protective boxes to the microbiological laboratory.

### 2.5. Bacterial Culture

The swab was inoculated under sterile thioglycollate broth tubes (5 mL) under aseptic conditions. The sample was allowed to incubate for 7 days at 36 °C. Subsequently, direct inoculation was always performed on blood agar and Mac Conkey agar. It was then placed in a thermostat for 24 h at 36 °C. Then, a qualitative reading of the grown bacterial colonies was performed and evaluated by a microbiologist. In the case of positive findings, more specific identification was performed, i.e., microscopy (Gram staining), isolation of pure culture or biochemical diagnostics. Pathogenic bacteria were further identified by specifying whether they were multiresistant strains [30,31]. Specifically, *Corynebacterium species*, Coagulase negative *Staphylococcus*, *Streptococcus* species, *Micrococcus* species and Sporulating microorganisms were identified using microscopy. More detailed identification for research purposes was not performed. *Acinetobacter* species and *Pseudomonas aeruginosa* were identified using biochemical diagnostics (biochemical wedge agar), *Enterobacter cloacae* (ESBL negative) and *Serratia rubidaea* (ESBL negative) were identified using enterotest and disk diffusion test (AmpC and ESBL Detection Set D68C) on Mueller–Hinton agar. *Enterococcus* species was identified using biochemical diagnostics (MEAT agar). *Staphylococcus aureus* (MRSA negative and positive) was identified using biochemical diagnostics (STAPHYtest), CHROMagar and Oxacillin Screen Agar 2 and 6. The result was interpreted as without a finding, i.e., without bacterial contamination, or with a finding, i.e., with bacterial contamination. The finding with bacterial contamination was further divided into the presence of nonpathogenic and pathogenic bacteria. The interpretation of the finding was also carried out in accordance with the valid legislation of the Czech Republic. This Act No. 306/2012 Coll. [32] says that objects and surfaces have to be used to treat patients after their decontamination.

### 2.6. Data Analysis

Research data were analyzed and processed in statistical software (TIBCO Statistica, version 12, Palo Alto, CA, USA). The statistical analysis was carried out in two phases. First, the classification of the first degree was performed on the basis of descriptive statistics (absolute and relative frequency). In the second phase, statistical tests were performed to determine significant relationships between indicators. For statistical testing of hypotheses, the test of agreement of two alternative distributions and the chi-square test with a significance level of *p* = 0.05 were used.

### 2.7. Ethical Considerations

This study did not include any ethically controversial issues; the research was not conducted on humans. The research was carried out in compliance with the Regulation of the European Parliament and of the Council No. 2016/679 of 27 April 2016. The research was carried out in accordance with the 1975 Helsinki Declaration and its most recent revisions, and in accordance with national ethical standards and regulations. The hospital and the individual workplaces agreed with the implementation of the research.

## 3. Results

### 3.1. Culture Results in Emesis Basins

For the purposes of the research, 11 nano emesis basins and 13 control emesis basins were used. A total of 132 swabs from nano emesis basins and 156 swabs from control emesis basins were taken throughout the research. Based on 132 (100.0%) swabs from nano emesis basins, there was a finding without bacterial contamination in 94 (71.2%) swabs and a finding with bacterial contamination in 38 (28.8%) swabs. Based on 156 (100.0%) swabs from control emesis basins, there was a finding without bacterial contamination in 104 (66.7%) swabs and a finding with bacterial contamination in 52 (33.3%) swabs (Table 1). The culture finding was represented by nonpathogenic and pathogenic bacteria.

### 3.2. Culture Results in Trays

For the purposes of the research, a total of 12 nano trays and 12 control trays were used, where 144 swabs from nano trays and 144 swabs from control trays were taken throughout the research. Based on 144 (100.0%) swabs from nano trays, there was a finding without bacterial contamination in 88 (61.1%) swabs and a finding with bacterial contamination in 56 (38.9%) swabs. Based on 144 (100.0%) swabs from control trays, there was a finding without bacterial contamination in 90 (62.5%) swabs and a finding with bacterial contamination in 54 (37.5%) swabs (Table 2). The culture finding in nano trays was represented only by nonpathogenic bacteria and in control trays it was represented by both nonpathogenic and pathogenic bacteria.

### 3.3. Culture Results in Boxes for Storing Medical Supplies

For the purposes of the research, a total of 3 nano boxes for medical supplies and 3 control boxes for medical supplies were used, where 36 swabs from nano boxes and 36 swabs from control boxes were taken throughout the research. Based on 36 (100.0%) swabs from nano boxes, there was a finding without bacterial contamination in 18 (50.0%) swabs and a finding with bacterial contamination in 18 (50.0%) swabs. Based on 36 (100.0%) swabs from control boxes, the findings were the same (Table 3). The culture finding was represented by nonpathogenic and pathogenic bacteria.

### 3.4. Evaluation of the Experiment

The research investigated whether there was a statistically significant difference (*p* ≤ 0.05) between bacterial contamination of nano and control objects and surfaces (emesis basins, trays and boxes for medical supplies) within 12 weeks. Based on the analysis, it was found out that no statistically significant difference was demonstrated between bacterial contamination of emesis basins (*p* = 0.407), trays (*p* = 0.808) and boxes for medical supplies (*p* = 1.000) in nano and control groups.

Further, it was investigated whether or not the nano emesis basins, trays and boxes for medical supplies would show bacterial contamination. Based on interval estimates (α = 0.05), it can be stated that already during the beginning of the monitoring period the relative degree of contamination in emesis basins was at least 0.152 (i.e., emesis basins will be contaminated in 15.2% of 66 swabs), in trays at least 0.289 (i.e., trays will be contaminated in 28.9% of 72 swabs) and in boxes for medical supplies at least 0.164 (i.e., boxes for medical supplies will be contaminated in 16.4% of 18 swabs). Furthermore, it was found out that in all research nano samples, bacterial contamination will always be detected in at least 26.5%, i.e., more than 1/4 of objects and surfaces will always be contaminated with bacteria.

The experiment also determined whether the degree of bacterial contamination (i.e., findings with bacterial contamination) and the method of treatment of selected objects and surfaces (objects and surfaces with and without the application of antibacterial and hydrophobic nanolayers) are independent. Based on the evaluation of the prevalence between the degree of bacterial contamination and the method of treatment of research nano and control samples, it was found out that a statistically significant dependence could not be demonstrated (α = 0.05; X^2^ = 0.210; *p* = 0.900).

Based on the above analyses, it was realized that no statistically significant difference (*p* ≤ 0.05) was found between the degree of bacterial contamination and the method of treatment or effectiveness of the nanolayer depending on the monitoring period. For this reason, a statistically significant incidence was investigated between the emesis basins, trays and boxes for medical supplies, regardless of whether they were treated with antibacterial and hydrophobic nanolayers. From the analysis of the data, it was found out that a finding with bacterial contamination in the emesin basins was in 31.3% (*N* = 288), in the trays in 38.1% (*N* = 288), in the boxes for medical supplies in 50.0% (*N* = 72) and in all research samples in 36.4% (*N* = 648). Based on the achieved level of significance, it can be stated that a statistically significant difference (*p* ≤ 0.05) was found between the degree of bacterial contamination of the emesis basin and the box for medical supplies (*p* = 0.003). In other cases, i.e., the dependence between the emesis basin and the tray (*p* = 0.083) and also the tray and the box for medical supplies (*p* = 0.067), it was not possible to prove a statistically significant difference.

## 4. Discussion

The provision of nursing care requires the use of various objects and surfaces intended for single or repeated use for the implementation of nursing interventions. In terms of the possible transmission of healthcare-associated infections, the problematic areas are mainly objects and surfaces that are intended for repeated use and which come into frequent contact with the hands of nurses. These objects can be contaminated with various bacterial agents, as confirmed by this and other studies [8,33]. An important aspect of preventing transmission of healthcare-associated infections through these objects and surfaces is the observance of the basic principles of mechanical cleaning and disinfection [6,8,18]. In the event that disinfection is not performed or is performed imperfectly, the agents of healthcare-associated infections may persist on the surfaces and may be involved in the transmission of infectious pathogens [12,13,14,15,16]. In this context, it is important to seek out and exploit new opportunities to minimize the risk of transmission of healthcare-associated infections through high-touch objects and surfaces used to provide nursing care.

This research, which was carried out in a regional hospital in the Czech Republic, verified the effectiveness of antibacterial and hydrophobic nanolayer, which was applied to selected objects and surfaces. The research revealed that the research samples with the applied antibacterial and hydrophobic nanolayer do not show better antibacterial activity compared to the research samples without surface treatments. However, the study did not investigate the initial bacterial burden on samples. Several studies [7,34,35] have evaluated the direct bacterial concentration on surfaces that have been plated without an enrichment phase. This surface treatment was very effective. These studies observed that most of the touch surfaces were contaminated and that the bacterial burden was highly variable between samples. This may explain the limitations of this study. Based on the research results, it was found that 28.8% of nano emesis basins and 33.3% of control emesis basins were contaminated with bacteria. The situation was similar for trays, when 38.9% nano trays and 37.5% control trays were contaminated with bacteria.

The results show that the applied nanolayer achieves low efficiency. This may have been due to testing in clinical conditions with possible external influences, but in laboratory conditions the effect of antibacterial and hydrophobic nanolayer was significant, especially on Methicillin-resistant *Staphylococcus aureus* [29]. Another possible influence may have been the dependence on the resistance of the surface treatment and damage to the layer during use. A limitation of the study may be that the activity of the nanoparticles was tested in laboratory conditions, not in medical facilities. As well as this, temperature and humidity can be limiting factors. It is also appropriate to consider a change in the composition and method of synthesis of the applied nanolayer. Efficacy could also be affected by the use of disinfectants. This shows that it is necessary to further develop and optimize the nanomaterial applied to individual objects and surfaces in terms of the results of this study. In future studies, it will be important to focus on identifying the negative circumstances that affect the effectiveness of this antibacterial nanolayer. It is also necessary to increase its antimicrobial efficacy. The method of application of the nanolayer can also be an influencing factor. Another study also states that the actual production processes, such as the thickness of the coating and the surface geometry, are also important for the essence of surface treatments. It also states that it is necessary to design and manufacture new polymeric and nanocomposite materials for effective use in healthcare [36]. The study [37] states that, when used, ZnO-C nanocomposite coatings are very effective for the elimination of *Pseudomonas aeruginosa*.

On the contrary, other studies [22,23,34,35,38] show that antibacterial surfaces, including modifications, are an effective way to prevent healthcare-associated infections. New self-disinfecting surfaces with heavy metal surfaces (copper, silver, etc.) have antimicrobial properties and can retain antimicrobial activity for several weeks to months [38]. For example, a study using copper surfaces revealed that the microbial load on copper objects was 73% lower than without any surface treatments [7]. Another study showed that the frequency of the contamination as well as the specific bacterial population bioburden is reduced on copper alloy surfaces [35]. Additionally, in the Salgado study [39] it was found that reduction of bacterial contamination was observed on objects with copper surfaces against objects without copper surfaces in 44%. For example, another study showed a small reduction of bacterial contamination on surfaces with copper [40].

This study showed that bacterial contamination of noncritical objects and surfaces is relatively high. In the case of emesis basins, all emesis basins were contaminated in 31.3% of 288 swabs. Trays for the preparation of injection and infusion material were contaminated in 38.1% of 288 swabs and boxes used for storing medical supplies were contaminated in 50.0% of 72 swabs. Another important finding is the fact that all research samples were contaminated in 36.4% of 642 swabs. The results also showed that the emesis basins were contaminated with pathogenic microorganisms, such as *Pseudomonas aeruginosa* (0.8%), *Acinetobacter species* (0.8%) or *Staphylococcus aureus* (1.3%). Pathogenic microorganisms were also detected on control trays. In this case, the presence of *Acinetobacter species* (0.7%), *Enterobacter cloacae* (0.7%) or *Serratia rubidaea* (0.7%) was detected. The presence of pathogenic bacteria was similar in boxes for storing medical supplies, such as Methicillin-resistant *Staphylococcus aureus* (2.8%) and *Enterobacter cloacae* (2.8%). The most common nonpathogenic bacteria were Coagulase negative *Staphylococcus* and Sporulating microorganisms. At the same time, it was found that the objects and surfaces were mainly contaminated with skin flora bacteria. Similar results have been found in other studies [1,8,11,12,13,14,41,42]. However, another study [43] points to the dangers of Coagulase negative *Staphylococcus*, which are associated with a high degree of antibiotic resistance and are among the main causes of bacteremia. If an infection occurs, treatment options are limited. The Ndegwa study [24] also showed that less than 50% of hospital items and surfaces and other portable medical devices were decontaminated appropriately using chemical disinfectants. This may be due to the fact that healthcare professionals may not adhere to the recommended exposure time or the prescribed concentration of the used disinfectant, including the decontamination procedure [38,44]. In this context, some studies mention that surface contamination of objects and surfaces is mainly due to human failure in the form of the absence of thorough cleaning and disinfection of surfaces rather than the use of an unsuitable product or process [33]. Thus, contaminated objects and surfaces have an effect on the possible cross-transmission of healthcare-associated infections [33]. When implementing nursing interventions, it is important to follow the principles of hospital hygiene, including decontamination and aseptic techniques during the performed procedures [44]. At present, there is still a consensus on the need to improve the mechanical cleaning and disinfection of surfaces, which are among the day-to-day essential elements of the effectiveness of the infection prevention program [38]. This study also found that some nurses do not adequately decontaminate objects and surfaces.

An important aspect of the prevention of healthcare-associated infections is the search for and optimization of options with surface treatment. Furthermore, it is important to optimize and adhere to the set hygienic-epidemiological principles, especially with a focus on the implementation of effective decontamination of objects and surfaces after their use [33,44]. At the same time, it is important to regularly check and search for clinically relevant sites of decontamination by various methods, e.g., using visual inspection, fluorescence, adenosine triphosphate bioluminescence, microbiological swabs and others [18,20,33]. There is also a need for continuous education and regular effective training of healthcare professionals in the prevention of infections associated with healthcare, including the implementation of simulation teaching methods. Studies have shown that educational interventions have a positive effect on performing decontamination of objects and surfaces [18,45,46]. Another clinically relevant issue is the knowledge of personnel, including nurses, who perform decontamination. The research has also shown that increasing knowledge of nurses is very important in some areas [47,48]. There are several aspects that affect the achievement of a high degree of efficiency in the decontamination of objects and surfaces, and traditional procedures and disinfection need to be increased and followed. At the same time, the cost-effectiveness of new technologies needs to be addressed [38]. At present, it is necessary to build on these results and compare the effectiveness of various prevention options with implementation in practice, while improving the quality and safety of healthcare services provided through education and the search for new options.

## 5. Conclusions

Prevention of healthcare-associated infections is an essential part of providing healthcare and nursing care. An important part of provision of healthcare is the use of decontaminated objects and surfaces to minimize the possible transmission of pathogens associated with healthcare to patients or medical staff. In some cases, decontamination of objects and surfaces is insufficient. For this reason, it is important to look for new possibilities for antimicrobial surfaces. Antibacterial and hydrophobic nanolayers may be one of the options. The effectiveness of the used antibacterial nanolayer is not confirmed in this research. Nano research samples show the presence of bacteria. However, there are study limits that could affect antibacterial efficacy. Based on the results of this research, it is important to optimize the applied nanolayer for its effectiveness. Some research states that some antibacterial copper surfaces may be effective in preventing healthcare-associated infections. Based on the results of the study, it is important to deal with the aspect of prevention of healthcare-associated infections and minimization of contamination of objects and surfaces using the current state of knowledge and implementation of new possibilities. It is important to note that approximately 1/3 of the items and surfaces used for nursing interventions may be contaminated with bacteria, including pathogenic bacteria. Based on the results of the study, it is necessary to strengthen the existing programs for the prevention of healthcare-associated infections and to use the available evidence to minimize the transmission of pathogens.

## Figures and Tables

**Table 1:** Culture results in emesis basins.

Nano			Control		
Category	Number of finding	%	Category	Number of finding	%
Without bacterial contamination	94	71.2	Without bacterial contamination	104	66.7
With bacterial contamination	38	28.8	With bacterial contamination	52	33.3
*p*-value of Nano versus Control emesis basins 0.407.					
Detailed analysis of culture finding from single swabs					
*Micrococcus* species	2	1.5	*Micrococcus* species	3	1.9
Sporulating microorganisms	5	3.7	Sporulating microorganisms	11	7.1
Coagulase negative *Staphylococcus*	27	20.4	Coagulase negative *Staphylococcus*	34	21.8
*Streptococcus* species	1	0.8	Coagulase negative *Staphylococcus*, *Micrococcus* species	1	0.6
*Enterococcus* species	1	0.8	Coagulase negative *Staphylococcus*, Sporulating microorganisms	1	0.6
*Pseudomonas aeruginosa*	1	0.8	*Staphylococcus aureus* (MRSA negative)	2	1.3
*Acinetobacter* species	1	0.8			

**Table 2:** Culture results in trays.

Nano			Control		
Category	Number of finding	%	Category	Number of finding	%
Without bacterial contamination	88	61.1	Without bacterial contamination	90	62.5
With bacterial contamination	56	38.9	With bacterial contamination	54	37.5
*p*-value of Nano versus Control trays 0.808.					
Detailed analysis of culture finding from single swabs					
*Micrococcus* species	3	2.1	*Corynebacterium* species	1	0.7
Sporulating microorganisms	23	16.0	*Micrococcus* species	3	2.1
Coagulase negative *Staphylococcus*	29	20.1	Sporulating microorganisms	16	11.1
Coagulase negative *Staphylococcus*, Sporulating microorganisms	1	0.7	Coagulase negative *Staphylococcus*	31	21.5
			*Acinetobacter* species	1	0.7
			*Enterobacter cloacae* (ESBL negative)	1	0.7
			*Serratia rubidaea* (ESBL negative)	1	0.7

**Table 3:** Culture results in boxes for storing medical supplies.

Nano			Control		
Category	Number of finding	%	Category	Number of finding	%
Without bacterial contamination	18	50.0	Without bacterial contamination	18	50.0
With bacterial contamination	18	50.0	With bacterial contamination	18	50.0
*p*-value of Nano versus Control boxes for storing medical supplies 1.000.					
Detailed analysis of culture finding from single swabs					
Coagulase negative *Staphylococcus*	10	27.8	Coagulase negative *Staphylococcus*	11	30.5
Sporulating microorganisms	7	19.4	Sporulating microorganisms	5	13.9
*Staphylococcus aureus* (MRSA positive, oxa-R)	1	2.8	Coagulase negative *Staphylococcus*, *Micrococcus* species	1	2.8
			*Enterobacter cloacae* (ESBL negative)	1	2.8

## Data Availability

The data are not publicly available.
